# Update on direct embryo transfer in sheep: hatched blastocysts increase conception rates

**DOI:** 10.3389/fvets.2026.1778321

**Published:** 2026-02-03

**Authors:** Ștefan Gregore Ciornei

**Affiliations:** Embryo Technology Laboratory, Faculty of Veterinary Medicine, "Ion Ionescu de la Brad" Iasi University of Life Sciences, Iasi, Romania

**Keywords:** conception rates, embryo, embryotransfer, hatched blastocysts, IVD, MOET, sheep

## Abstract

This article presents an overview of the update on direct embryo transfer using hatched blastocysts. Recent research has observed differences in conception rates in sheep following surgical transfer. All sessions included in the study followed the same standard protocol, resulting in the creation of two groups, with the control group (CG) being the one in which blastocysts were transferred, and the experimental group (EG) received hatched blastocysts. Embryos were obtained from meat sheep through *in vivo* derived (IVD) and transferred to crossbred sheep synchronized with very obvious corpus luteum (CL) on at least one of the ovaries (84.21%). Thus, a retrospective study highlights the clear success of embryo recipients who also received hatched blastocysts (code 9.1) compared to recipients with blastocysts only (code 6.1, 7.1). The embryo recovery rate at 6.5 days, determined by laparoscopic uterine flushing, was 84.3%. In terms of the quality of the embryos obtained, over 74.5% were transferable (not statistically significant, 76.6% in Suffolk and 72.3% in Ille de France), and over 12% of the embryos hatched. The study found that the pregnancy rate in recipient ewes receiving code 9.1 embryos (expanded blastocysts) through direct IVD transfer during the breeding season was 86.9%. These findings, when compared to previous research, highlight the potential for further exploration and innovation in this area. Nonetheless, it is important to note that there is a scarcity of literature addressing the direct transfer of IVD embryos with expanded blastocysts.

## 1 Introduction

The global expansion of small ruminant breeding over the last few decades has been significantly bolstered by the advancement and refinement of assisted reproductive technologies (ART) (1). While certain ART methods such as estrus induction, estrus synchronization (2), sperm production and quality control (3) and artificial insemination (AI) are widely used, the uptake of other techniques, including multiple ovulation and embryo transfer (MOET), *in vitro* embryo production (IVEP), and embryo cryopreservation, remains limited in sheep, in their routine application (4). The physiological response to stress, environment, and climate is also a factor that must be taken into account (5).

*In vivo* embryo production efforts in small ruminants trace back to the 1930s, with the first successful documented case occurring in 1934 (6). Since that time, most embryo recovery and transfer procedures have historically relied on surgical methods. Essentially, embryo collection in small ruminants can be achieved through surgical, laparoscopic, or transcervical approaches (7, 8). The laparotomy technique allows for accurate counting of the number of corporpus luteum (CLs) and assessment of the total embryo recovery rate (RR). However, the disadvantages are the relatively high cost of surgical equipment, stress for the animal, and consequences due to manipulation of the uterine horns (9). The primary constraint preventing the wider use of embryo collection and transfer in the small ruminant industry today is the reliance on surgical interventions, which carry inherent drawbacks related to cost, animal welfare, and restricted repeated use.

According to the International Embryo Transfer Society (IETS) Data Retrieval Report, the total sheep embryo production both *in vivo* derived (IVD) and *in vitro* produced (IVP) has increased over the last 8 years (10). Transferring multiple embryos to reduce the number of recipient ewes is economically more sustainable. Conversely, other available data have shown that increasing the number of embryos transferred resulted in a decrease in the number of newborn lambs (11, 12).

A positive correlation was found between developmental stage of transferred embryos and pregnancy rates (13). Usually, embryos are transferred at late morula, early blastocyst or expanded blastocyst stages (14). The embryos are evaluated in Petri dishes with small wells, with variable magnification, and from multiple directions by turning them over. Grading is performed after collection and before transfer to the synchronized recipient female.

The size of sheep embryos is 150 to 190 μm, including a zona pellucida thickness of more than 10 μm. The cleavage and development of embryos in small ruminants, up to the hatched blastocyst stage, takes between 6–6.5 days. An ideal embryo is compact and spherical, blastomeres are of similar size with even color and texture, cytoplasm is not granular or vacuolated, the perivitelline space is clear and contains no cellular debris, and the zona pellucida is uniform, not cracked or collapsed, and without debris on its surface.

The objective of this study was to evaluate the effects of embryo development stage (hatched blastocyst) and embryo quality (grades 1 and 2) at the time of collection (on day 6.5) on the conception rate after transfer to mixed recipient ewes. Following routine IVD protocols in sheep, collections of hatched embryos were observed using the surgical method, which generated frequent pregnancies, leading to the motivation for this retrospective study.

## 2 Methods

The present study was conducted during the normal breeding season, carried out in the same laboratory (Embryotechnology Laboratory by USV Ias, Romania) led by the same highly qualified personnel, over several years (2017–2024).

This study was approved by the Iasi University of Life Sciences (IULS), Faculty of Veterinary Medicine, Bioethics committee following the EU 2010/63 and National directives Ord. 28/31–08–2011 and National Law 206/2004.

### 2.1 Embryo production

Procedures for obtaining embryos through MOET in meat sheep (Suffolck and Ille de France) were selected, following which embryos in stage 9 of development and class 1 quality (code 9.1) were collected, and traceability and conception rates upon transfer were monitored.

**Figure 1:**
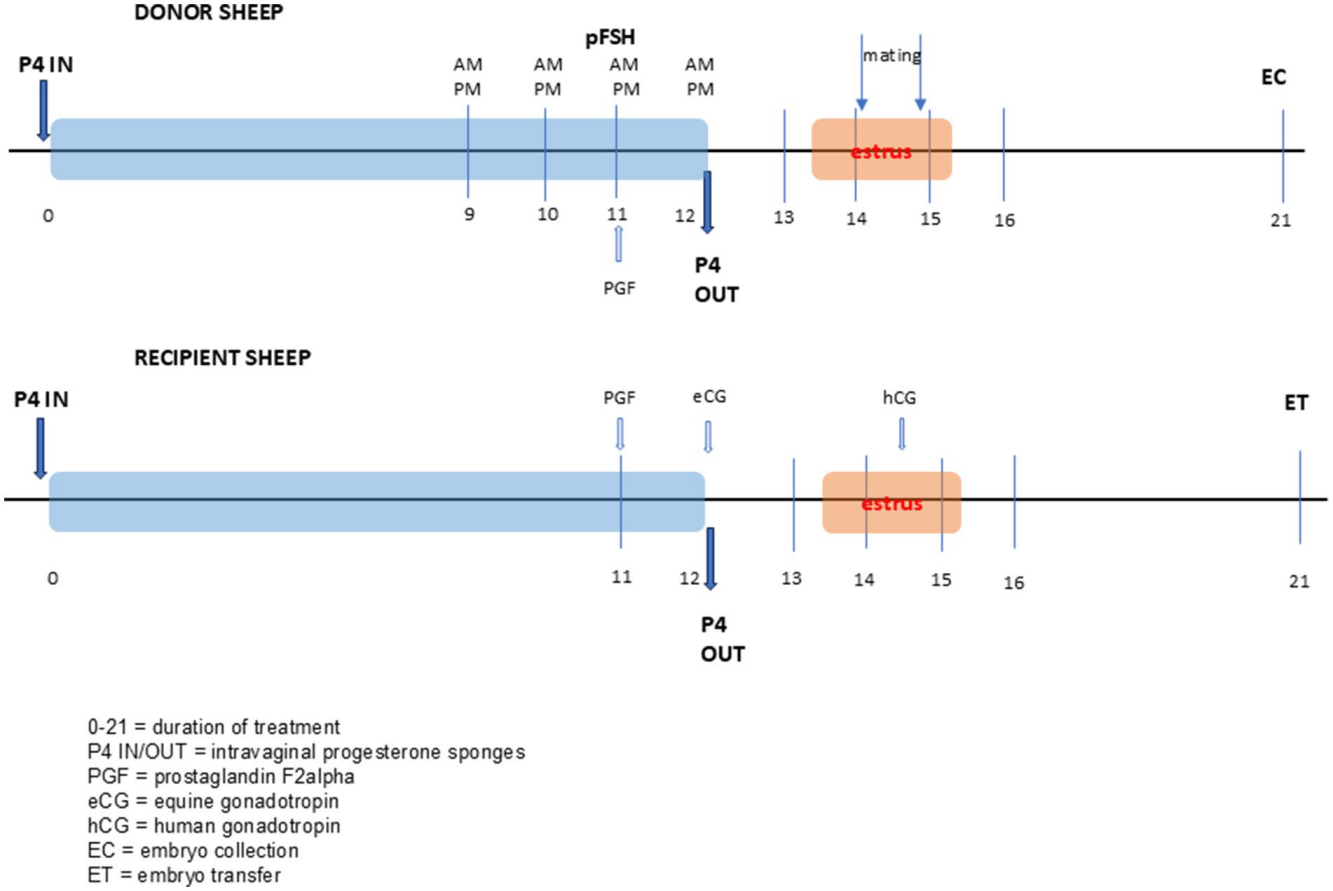
Therapeutic protocol for ovarian hyperstimulation in donors and synchronization of recipients.

The polyovulation treatment of donors was classic, used with good results in this laboratory (12, 15), using P4 vaginal sponges (Chronogest, MSD, Netherlands) for 12 days, on day 11 a dose of PGFanalogue, cloprostenol in 125 μg dose (Estrumate, MSD, Netherlands), and from day 9 until the day of removal, pFSH (Pluset, Calier, Spain), was administered twice a day in decreasing doses (2/1.5 cc; 1.5/1.5 cc; 1/1 cc; 1/0.5 cc), for a total of 500 IU. At 36–55 h, at the time of estrus, two matings were organized at 12-h intervals, followed by embryo collection 6.5–7 days after mating (Figure 1).

### 2.2 Recipient management

Recipients were selected based on age, general health, and body condition score (BCS). Local primiparous cross-breed ewes (not wool ewes) were synchronized using a P4-PGF-eCG protocol (Figure 1). The ewes received a 20 mg FGA intravaginal sponge (Chronogest) for 12 days, and cloprostenol equivalent to 125 μg PGF2α (Estrumate) on day 11. On day 12, when the intravaginal sponge was removed, ewes received an 200 IU eCG (Folligon, Intervet, Holland). And on day 14.5 a dose of 400 IU of hCG (Chorullon, Intervet, Holland). Oestrus detection was performed with test rams between 30 and 60 h after PGF administration. Embryo transfer was performed 6.5 days after oestrus detection.

Body condition score (BCS) was assessed when selecting donors and recipients, all of whom were included with a score of 4. Ultrasound was performed with a Honda 5 MHz transrectal probe 30 days after transfer.

### 2.3 Preparation of recipients and donors for surgery, uterine flushing

Donors and recipients were not allowed to eat for 18 h before surgery, while water removal was done 12 h before. Donors and recipients were premedicated with a suitable epidural anaesthesia, and also local anaesthesia along the incision line (60 mg procaine hydrochloride, Procarom 2% Romvac, Romania), and non-steroidal anti-inflammatory (flunixin 1.5 mg/kg, intramusculary on the day of embryo transferMegadyne, Virbac, India), given well in advance of any procedure. The ewe was placed in dorsal recumbency on a specially designed laparoscopy cradle. Uterine flushing was performed using Vigro complete flush (Vetoquinol, USA), a two-way catheter (Vortech 8 Ch), and a filter (EmSafe Filter). In order to improve estrous behavior and ovulation the presence of teaser males was permitted from the moment of intravaginal device removal until 60 h later.

### 2.4 Embryo evaluation, accurate assessment of ovulation, and embryo transfer

The recovered fluid was examined under 20–100 × magnification using a stereomicroscope, under sterile and isothermal conditions. In sheep, embryo development occurs more rapidly and the blastocyst stage is reached 0.5–1.0 days earlier than in cow embryos (International Society for Embryo Technology Manual, 5th edition) (10, 12).

**Figure 2:**
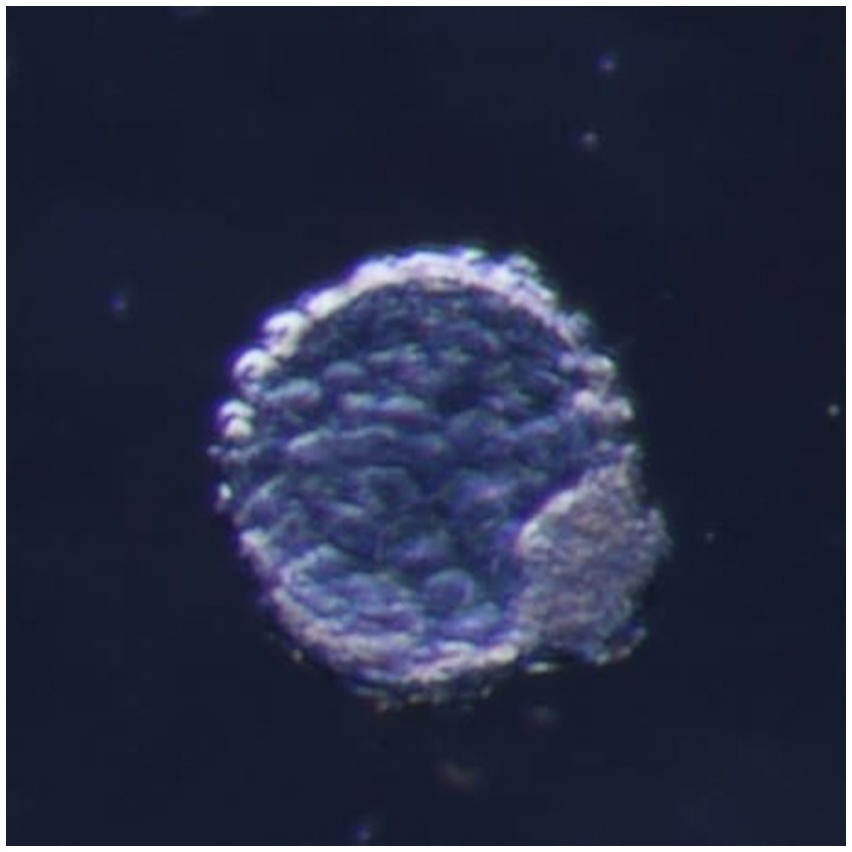
Hatched blastocyst code 9.1, obtained by flashing 7.5 days after estrus, magnification 100, dark background.

Excellent and good embryos (code 1 and 2) were loaded into the Tomcat catheter in a small volume of holding medium between two air bubbles (for transfer to the control group of sheep). They were also aspirated separately, along with hatched blastocysts with one unhatched code 1 embryo (for the experimental group) (Figure 2).

**Figure 3:**
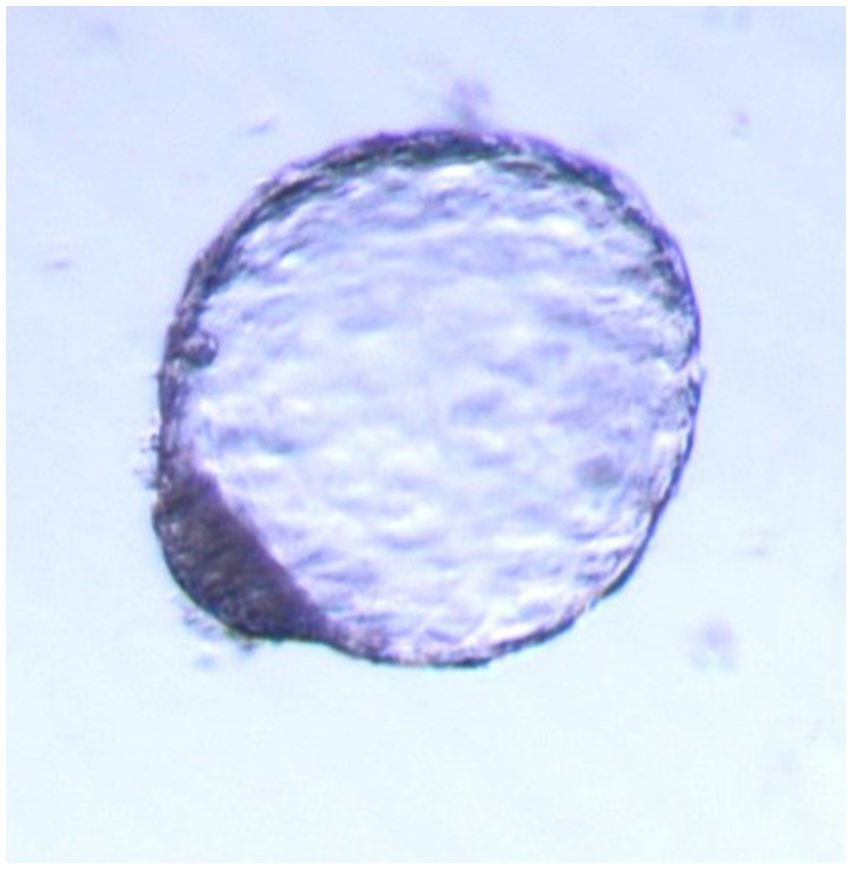
Hatched blastocyst code 9.1, obtained by flashing 7.5 days after estrus, magnification 100, bright field. No zona pellucida observed, compact blastomeres, uniform trophoblast, compact ICM with evident cells.

The perfect synchronization of the recipients with the embryo was assessed only at the time of laparotomy and macroscopic examination of the ovaries. The recipient ewes with a well-defined corpus luteum received embryos in the uterine horn ipsilateral to the ovary with CL. ET in the recipients’ group was performed by a minimally invasive surgical method (Figures 2, 3).

Two groups of recipient ewes were thus organized: the control group (CG) of 31 ewes that received embryos code 1 and 2, and the experimental group (EG) of 23 ewes that received embryos code 1 and hatched blastocysts (9.1) (Table 1).

**Table 1:** Production and origin of embryos, conception rate in recipients, depending on the stage of blastocysts.

Donor sheep	Poliovulatory response			Embryonic production/quality		
	Average CLs/donor	R ovary	L ovary	Recovery rate (RR%)	Code 1 + 2 transferable	Code 3 + 4 nontransferable
Ille de France	29	46.9% *^a^*	53.1% *^b^*	86.2%	76.6%	23.4%
Suffolk	31	35.6% ^c^	64,4% ^d^	82.4%	72.3%	27.7%
Average	30	41.3% *^e^*	58.7% *^f^*	84.3%	74.5%	25.5%
The Chi-Squared test. *a-f* -represents statistically insignificant differences (*p* > 0.05), *a/b* = 0.5774, *c/d* = 0.1060, *e/f* = 0.1214						

**Embryo recipients, non-wool-producing shep**	**C.G** **(control group)**	**E.G** **(experimental group)**
Embryo quality/no/transfer	2 x embryos code 1 or 2	1 x embryo code 1 1 x embryo 9.1
Hatched blastocysts	no	yes
No recipients	31	23
Positive pregnancy ultrasound	19	20
Conception rate (CR%)	61,3 *^g^*	86,9 *^h^*
*p* value. The Chi-Squared and ANOVA/T-test. *g/h*	*p* = 0.0379 The results are identical confirming a statistically significant difference (*p* > 0.05).	

### 2.5 Statistical analyses

Data on ovulation, conception rates, within groups were compared using chi-square and ANOVA tests, and a value of *p* < 0.05 was considered statistically significant. Statistical analysis was performed using Prism version 8 (GraphPad 5.0 software. La Jolla, CA, USA, www.graphpad.com, accessed on November 21, 2025).

## 3 Results

Embryo production. After applying the P4-12 days-Pluset 500 mg treatment, positive values of follicular hyperstimulation and ovulation were obtained in donor meat sheep. An average ovulation of 30 corpus luteum was obtained, with variations between 23 and 33 CL. It is noteworthy that in both breeds there is a persistent predilection for hyperstimulation and average polyovulation for the left ovary (58.7%) compared to the right ovary (41.3%), but this is not statistically significant (*p* = 0.1214). For the left ovary, an average of 1.7 CL more than on the right ovary was obtained (Table 1).

The embryo recovery rate at 6.5 days, by laparoscopic uterine flushing, was 84.3%, and in terms of the quality of the embryos obtained, over 74.5% were transferable (not statistically significant, 76.6% in Suffolk and 72.3% in Ille de France). The transferable embryo category consists of embryos rated as excellent and good (codes 1 and 2), while the non-transferable embryo category includes unfertilized oocytes (UFO), degenerated or dead embryos, and quality codes 3 and 4 (25.5%). Flushing performed on ewes more than 6.5 days after estrus generated over 12% of the hatched embryos (codes 8–1 and 9.1) used in this study (Figure 2).

Recipient management. Synchronizing receipent with a body score of 4 during the breeding season using the P4-12-eCG-hCG method is most effective because it induces estrus in 100% of ewes and induces and groups ovulation in 84.21% of cases, with high-quality CL.

Embryo transfer and group formation fecundity. Transrectal echography 30 days after ET confirmed the diagnosis of pregnancy. The experimental group (EG) achieved a conception rate (CR) of 86.9% (20/23) compared to the control group (CG) of 61.3% (19/31). The difference was statistically significant *p* = 0.0379 (*p* > 0.05) where expanded blastocysts were transferred.

## 4 Discussion

Embryo transfer in sheep is a challenge in the ruminant industry because it is limited by certain anatomical and physiological considerations such as the small size of ruminants, the particularities of the cervix, seasonality, and ovarian reactivity to hyperstimulation, as well as the additional costs associated with surgical procedures. Therefore, any result that improves the technique and, above all, the conception rate is welcome (4).

In a direct ET protocol, it is mandatory for donor ewes to be synchronized with recipients; basically, we are more interested in ovulation synchronization. Donor ewes always produce embryos using the MOET method, and their age is around 6 days. Recipients must be synchronized with donors on day 6–6.5 so that, at the time of ovulation (12 h before the end of estrus, i.e., day 6), most of them are at the same stage of estrus expression (9, 12, 16).

The theoretical results refer to the synchronization of ovulation in three directions: inducing polyovulation in donors, inducing ovulation in recipients, and synchronizing ovulation in as many recipients as possible. Therefore, synchronizing the expression of estrus and anticipating ovulation is essential (4).

Multiple ovulation and embryo transfer (MOET) technologies have contributed and continue to make substantial genetic progress in sheep in several countries around the world. The effect of the size and breed of recipients on embryo survival was studied by Naqvi et al. (17). They investigated developmental competence, birth and survival of lambs after transfer of two or three embryos of a small prolific breed into large size non-prolific recipient ewes. The results showed that recipient breed did not affect survival and weaning performance of lambs from the prolific breed (18). In the present study, embryos similar in prolificacy (meat breeds) were successfully transferred to less prolific local breed recipients. The results showed a very good implantation and lambing rate.

Transfer at earlier or later stages does not bring significant advantages and does not reduce viability to term. In fact, while embryos derived *in vivo* at the late morula and expanded blastocyst stages resulted in comparable pregnancy rates, lower pregnancy rates were achieved with the transfer of hatched embryos (19). The data obtained by us in this study improve the outlook for the innovative future of the IVD industry for sheep.

Embryos transferred at earlier stages (2–3.5 days post-fertilization) resulted in acceptable pregnancy rates (13), but without a practical advantage, as viability does not change compared to later embryonic stages (14).

The correlation between embryo morphology and pregnancy rates has been discussed for many years, but there is still a need for accurate assessment of embryo quality classification prior to transfer, and in most cases, quality scores are the result of experienced practitioners (20).

Currently, embryos are assessed according to IETS manual 2025, the stage of embryo development is classified using a numeric scale, ranging from 1 to 9 (from unfertilized oocytes to expanded hatched blastocysts, respectively). Embryo quality is evaluated under a numerical code based on morphological integrity of embryos, ranging from Code 1 to 4 (from excellent or good to dead or degenerated embryos). Generally, unless otherwise agreed, Code 1 to 3 are considered as transferable embryos when used fresh (12, 15, 21).

Similar research to this study was published by King (22), where one or two transferable embryos were transferred to each of the 256 synchronized recipient ewes, and pregnancy diagnosis was performed on day 36 after embryo transfer. Embryos at the hatched blastocyst stage had higher viability *in vivo* compared to embryos at the late morula stage (59.0 ± 10.6% vs. 36.2 ± 9.7%; *p* = 0.083). The viability of grade 1 embryos was higher than that of grade 2 embryos (53.6 ± 7.8% vs. 35.9 ± 10.2%; *p* < 0.05) (22). These results reinforce and validate the theory that hatched embryos have a more pronounced tendency to produce a pregnancy (23).

Similar studies in humans, Kim (24) show the same tendency for hatched blastocysts to result in higher pregnancy rates, even after thawing (15.7% for one and 19.5% for two embryos). Therefore, the transfer of hatched blastocysts could be considered a superior method in human IVF practice (24).

However, an important limiting factor that continues to affect the success of ET-IVD programs is the variability in ovarian response and embryo yield (25), embryo quality, recipient uterine environment, and elimination of risk factors leading to embryo mortality (9, 15).

It is known that stress after conception leads to increased corticosterone levels, which can inhibit embryonic development and reduce the number of cells in the inner cell mass, compromising embryo quality and affecting pregnancy rates (26, 27).

Pregnancy losses in ungulate species occur mainly in the second week of gestation, when the embryo undergoes a series of processes of differentiation, proliferation, and cell migration, collectively referred to as conceptus elongation (28). To this end, a series of management actions such as avoiding stress, ensuring optimal feeding and housing conditions, as well as medication such as the administration of NASD (which reduces inflammation and prevents PGF synthesis), the administration of P4 or CL trophicisation through the injection of GnRH and hCG, lead to the prevention of embryonic mortality (15).

A well-developed CL with an obvious outer corona will generate significant progesterone secretion (12, 23), confirmed by studies showing that conception rates were lower in recipients with progesterone levels below 1 ng/mL compared to those with levels above 3 ng/mL (29).

Similar pregnancy rates were reported by Dattena (30), and of the 32 blastocysts derived *in vivo* and transferred fresh, 26 lambs (81.2%) were born. The pregnancy rate after transfer of fresh IVP blastocysts was lower (*p* < 0.07) than that of in vivo embryos (54.3% vs. 90.0%, respectively), reported Papadopoulos (31).

## 5 Conclusion

The study found that the pregnancy rate in recipient ewes receiving code 9.1 embryos (expanded blastocysts) through direct IVD transfer during the breeding season was 86.9%. These findings, when compared to previous research, highlight the potential for further exploration and innovation in this area. Nonetheless, it is important to note that there is a scarcity of literature addressing the direct transfer of IVD embryos with expanded blastocysts.

## Data Availability

The datasets presented in this study can be found in online repositories. The names of the repository/repositories and accession number(s) can be found in the article/supplementary material.
